# Identifying susceptibility genes by using joint tests of association and linkage and accounting for epistasis

**DOI:** 10.1186/1471-2156-6-S1-S147

**Published:** 2005-12-30

**Authors:** Joshua Millstein, Kimberly D Siegmund, David V Conti, W James Gauderman

**Affiliations:** 1 National Oceanic and Atmospheric Administration/National Marine Fisheries Service, Alaska Fisheries Science Center, 7600 Sand Point Way NE, Seattle, WA 98115 USA; 2 Keck School of Medicine of the University of Southern California, Department of Preventive Medicine, Division of Biostatistics, 1540 Alcazar St., CHP suite 220, Los Angeles, CA 90089 USA

## Abstract

Simulated Genetic Analysis Workshop14 data were analyzed by jointly testing linkage and association and by accounting for epistasis using a candidate gene approach. Our group was unblinded to the "answers." The 48 single-nucleotide polymorphisms (SNPs) within the six disease loci were analyzed in addition to five SNPs from each of two non-disease-related loci. Affected sib-parent data was extracted from the first 10 replicates for populations Aipotu, Kaarangar, and Danacaa, and analyzed separately for each replicate. We developed a likelihood for testing association and/or linkage using data from affected sib pairs and their parents. Identical-by-descent (IBD) allele sharing between sibs was explicitly modeled using a conditional logistic regression approach and incorporating a covariate that represents expected IBD allele sharing given the genotypes of the sibs and their parents. Interactions were accounted for by performing likelihood ratio tests in stages determined by the highest order interaction term in the model. In the first stage, main effects were tested independently, and in subsequent stages, multilocus effects were tested conditional on significant marginal effects. A reduction in the number of tests performed was achieved by prescreening gene combinations with a goodness-of-fit chi square statistic that depended on mating-type frequencies. SNP-specific joint effects of linkage and association were identified for loci D1, D2, D3, and D4 in multiple replicates. The strongest effect was for SNP B03T3056, which had a median *p*-value of 1.98 × 10^-34^. No two- or three-locus effects were found in more than one replicate.

## Background

The need to account for gene × gene interactions in the search for susceptibility genes for complex diseases such as cancer, diabetes, hypertension, and obesity has been widely suggested. However, accounting for interactions is not a trivial task due to the serious problem of multiple testing created by the large number of possible interactions even for a relatively small set of candidate genes. This problem is compounded by the notoriously low power of formal tests for interaction. In the context of modeling association in unrelated individuals, Devlin et al. [1] proposed a testing strategy that conserves power by jointly testing main effects together with interactions and by adjusting for multiple tests by controlling false discovery rates (FDR). However, this method suffers from interpretability difficulties because a positive test of a set of main effects and interactions for several loci does not necessarily imply that all loci in the set are contributing to the significance of the test. Also, the method requires exhaustively testing sets of two and possibly three genes. We propose an analytic strategy that uses likelihood ratio tests (LRT) within a framework to test main effects and interactions of association and/or linkage jointly, conditional on significant single-locus effects. This strategy also incorporates a screening statistic that reduces the number of marker combinations that need to be tested for multilocus effects.

To apply this testing framework to nuclear family data, the investigator must choose a particular type of test. An important advantage of conditional-on-parental-genotype (CPG) transmission disequilibrium test (TDT) methods, for testing association due to linkage disequilibrium (LD), over unconditional methods is that they are not subject to confounding due to population stratification. Generally, these methods are able to be implemented using standard statistical software packages. However, if there is more than one affected offspring per family, directly applying the TDT by treating the offspring as if they are from independent families will no longer provide a valid test of association when there is linkage. This is due to a downward bias in the standard error estimator for the association parameter [2,3]. We propose a model for the CPG likelihood that can be fit using standard statistical software and can be used for joint tests of linkage and association. We apply our proposed testing framework to single-nucleotide polymorphism (SNP) data in disease regions and non-disease regions from the simulated Genetic Analysis Workshop 14 (GAW14) data to demonstrate the performance of this approach in the context of a candidate gene study.

## Methods

### Data

Nuclear family data were extracted from the first ten GAW14 simulated replicate datasets. Kofendrerd Personality Disorder (KPD) disease status and genotype data were used for the first two affected sibs in each nuclear family, but only genotype data were used for the parents. For each replicate, there were 100 affected sib-pair-parent nuclear families that were obtained from each of the populations Aipotu, Kaarangar, and Danacaa with no missing data. All 48 SNPs from the six disease regions were included in the analysis as well as 10 SNPs from two regions on chromosomes 2 and 8 that were simulated with LD but had no relation to disease.

### Analytic approach

Let *g*_1_, *g*_2_, *g*_*m*_, *g*_*f *_denote the genotypes of two affected offspring, mother, and father at a marker locus under study, and let *D*_*i *_denote the disease status of offspring *i*. The CPG likelihood for an individual family takes the form *P*(*g*_1_, *g*_2_|*g*_*m*_, *g*_*f*_, *D*_1_, *D*_2_) [4]. From the conditional laws of probability the likelihood can alternatively be written as *P*(*g*_1_|*g*_*m*_, *g*_*f*_, *D*_1_, *D*_2_) × *P*(*g*_2_| *g*_1_,*g*_*m*_, *g*_*f*_, *D*_1_, *D*_2_). The basic analytic approach, developed by Millstein et al. [5], is based on the approach described by Self et al. [6] for estimating association due to LD in case-parent-trios. By making the reasonable assumption that *D*_2 _without *g*_2 _provides no information on *g*_1 _conditional on D_1_, the first part of the product can by modeled using the standard CPG conditional logistic regression approach, i.e.,



where *G*_*i *_denotes an indicator variable for alleles at *g*_*i *_and exp(*β*) is an association estimate of relative risk for the genotype. The second factor of the product can be modeled by including a covariate, *e*_*ij*_, to indicate the expected number of alleles shared identical by descent (IBD) by affected sibs *i *and *j *given the genotypes of both sibs and their parents. The resulting likelihood for both sibs would take the form



where the sums with respect to g* are over all possible offspring genotypes given the parental genotypes, and *e*_1_* is the expected IBD allele sharing between sib 1 and pseudosib * given the genotypes of both sibs and their parents, and *γ *is a measure of linkage. This model can be easily fit using the SAS (SAS Institute Inc., Cary, NC) procedure PROC PHREG with a conditional logistic regression approach, by creating a risk set for each affected sib and creating a covariate indicating IBD allele sharing for all members of the sib 2 risk sets (this covariate is set to zero for members of the sib 1 risk sets). The choice of which sib to assign the sib 1 position does not affect the likelihood. This approach can be used to test the null of no association in the presence of linkage (H_0_: *β *= 0 and *γ *≠ 0), linkage (H_0_: *γ *= 0), or joint association and linkage (H_0_: *β *= 0 and *γ *= 0). Among the advantages of using this approach are 1) it is easy to implement using standard statistical software; 2) direct adjustment for individual level covariates (for effect modification) is possible; 3) LRTs can be used to test jointly for multi-locus effects of association; 4) LRTs of gene × environment interactions are possible (see below); 5) joint LRTs of linkage and association are possible; 6) IBD sharing is explicitly modeled.

### Testing framework

**Figure 1:**
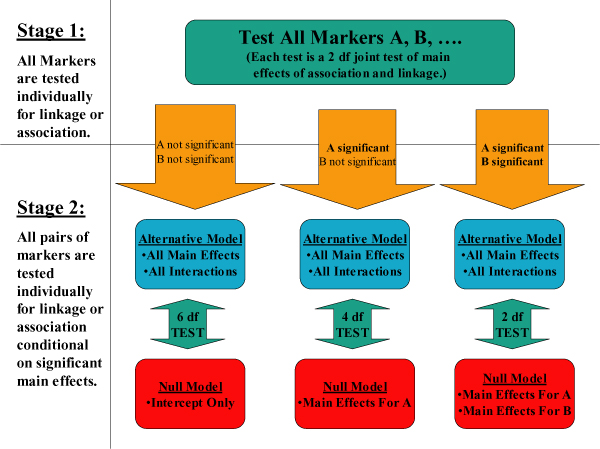
**Interaction testing framework flowchart for two stages. **The flow chart exhibits the first two stages of the testing framework. If tests of individual markers achieve statistical significance in the first stage, then the main effect terms for these markers are included in unsaturated (null) models in second stage likelihood ratio tests.

We employ a testing framework in which LRTs are performed in stages that are determined by the highest order interaction term in the saturated model and multilocus effects are tested conditional on significant lower order effects. Considering two loci, A and B, the linear predictor for the saturated model would be *β*_A_G_A _+ *β*_B_G_B _+ *β*_AB_G_A_G_B _+ *γ*_A_e_12 _+ *γ*_B_f_12 _+ *γ*_AB_e_12_f_12_, where e_12 _and f_12 _are the IBD covariates for loci A and B, and *β*_AB _and *γ*_AB _are interaction parameters for association and linkage, respectively. For two stages of testing, i.e., involving the main effects and the first order interactions in the preceding linear predictor, the testing would be conducted as shown in Figure 1. The third stage tests are performed in an analogous manner for all three-locus combinations, i.e., tests involving second order interactions are conditioned on main effects and first order interactions that were significant in the first and second stages.

We restricted our investigation to main effects and interactions between two or three SNPs in different chromosome regions, thus our focus was on gene × gene interactions rather than haplotype effects or within-gene SNP interactions. We modified the testing framework by prescreening SNP combinations using a mating type screening statistic (MS), and we tested only those combinations with an observed MS above a cut-off value.

### Screening statistic

If loci interact to produce disease in an offspring, then mating types that are likely to produce the susceptibility multi-locus genotype will be present in the parents of cases more likely than we would expect based on marginal mating type frequencies. For a diallelic locus, there are six possible parental mating types if we ignore parent-of-origin effects, AA × AA, AA × Aa, AA × aa, Aa × Aa, Aa × aa, aa × aa, and thus 36 possible mating types for a pair of loci or in general, 6^*k *^possible mating types for *k*-locus genotypes. Let *m*_*i *_denote the number of parental pairs that are described by the *i*^th ^multi-locus mating type and let *r *denote the number of possible mating types. Then the statistic,



which is equivalent to a goodness-of fit chi-squared statistic, can be used to screen gene combinations for case-parent designs thus reducing the number of gene sets that require testing for multi-locus effects. The quantity *E*[*m*_*i*_] is calculated from the observed single-locus mating type frequencies by assuming independence between loci in the population, i.e., the power of the method will be decreased if the loci are in LD in the population. In these analyses, the combinations of markers analyzed involved loci on separate chromosomes. The MS statistic uses only between-mating type information, which is independent of the within family information that is used in any CPG TDT analysis [6-9].

## Results and Discussion

**Figure 2:**
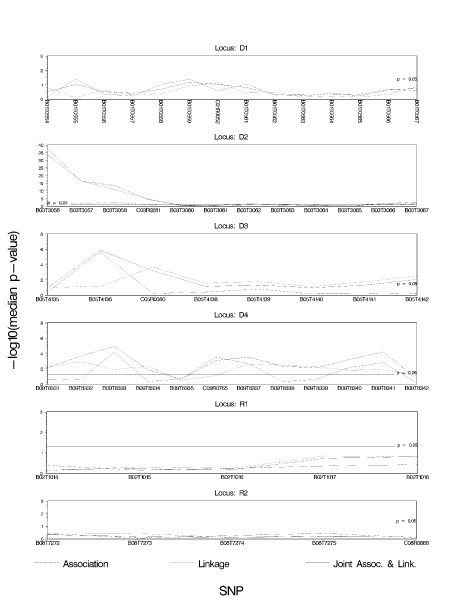
**Association, linkage, and joint tests of association and linkage. **Negative log_10 _of the median *p*-values are presented for analyses of the first 10 replicate simulations from the GAW14 data. Main effects of association, linkage, and joint linkage and association are shown for six chromosomal regions, four related to the KPD phenotype, D1–D4, and two regions, R1 and R2, that are in LD but are not related to KPD.

The FDR was controlled at the significance level *α *= 0.05 per replicate by allocating *α *= 0.017 to each of three testing stages and controlling FDR within each stage. Significant tests for stage 1 marginal SNP effects demonstrate that joint effects of linkage and association were detected in the four disease regions, D1–D4, despite heterogeneity in the definition of KPD between populations (Table 1). However, significant effects were detected in only 2 of the 10 replicate datasets for region D1 and no significant effects were found for the effect modifying SNPs at loci D5 and D6, C10R0880 and C02R0097. The lack of LD in region D1 explains the absence of an association signal but not the lack of a linkage effect (Figure 2), which may be attributed to the low frequency of the disease allele (0.015 from GAW14 Answers). In region D2, haplotypes were sorted as a character string (from left to right) and the disease allele was defined to be located on adjacent haplotypes after sorting (as stated in the answers). Thus, we should expect SNPs on the left to be in strongest LD with the disease allele. In fact, our first stage results show a strong decreasing association signal over four SNPs, starting from the left of region D2 (Figure 2). Within regions D3 and D4, disease-carrying haplotypes were chosen by similar frequency, thus we should not expect LD between the disease allele and SNPs within the region to depend on SNP location but rather on association with susceptibility haplotypes. Compelling evidence of both linkage and association is apparent in disease regions D2, D3, and D4 (Figure 2).

The solid line in Figure 2 representing the joint test of linkage and association generally lies above (more significant) the lines for the independent tests of association and linkage, which implies that the power of the joint test under these conditions is greater. A highly significant *p*-value due to association occurs at SNP B03T3056, whereas the linkage effect is non-significant at this SNP. This pattern is consistent with the observation that a linkage effect would not be expected conditional on association if the actual disease SNP is included in the analysis [10]. Although SNP B03T3056 is not the disease causing SNP, it may be so strongly in LD with the true disease SNP that there is essentially no conditional linkage effect.

The MS statistic was used to restrict the investigation to the top 20% of two-locus combinations and the top 10% of three-locus combinations. No two-locus effects were detected, and no three-locus effect was sufficiently strong to be detected in more than one replicate dataset after controlling the experiment-wise FDR at 0.05. Relaxing the significance criteria did not yield consistent effects across replicates. The multi-locus effect involving {C01R0052, B03T3056, C05R0380} was identified in replicate 1 and the effect involving {B03T3062, B09T8341, B02T1017} was identified in replicate 2 (Table 1). The test of the multilocus effect of {B03T3062, B09T8341, B02T1017} was significant after conditioning on the marginal main effect of B09T8341, and {C01R0052, B03T3056, C05R0380} was significant conditional on B03T3056. Thus three of these five disease-related SNPs were identified by their involvement in multi-locus effects in these replicates, and one SNP, B02T1017, was falsely identified as a disease-related SNP. Latent phenotype P1 was the result of a D1, D2 interaction and latent phenotype P2 involves a D2, D3 interaction, therefore the observed {D1, D2, D3} multilocus effect is consistent with the simulation design. The D1, D4 interaction has a penetrance of 1.0 for phenotype P3, thus the {D1, D4, non-KPD region 7} effect could be partially explained by that interaction.

The lack of significant two-locus effects together with the lack of consistency for the three-locus effects indicates a general lack of power under these conditions for detecting multilocus effects conditional on significant marginal effects after accounting for multiple tests even after pre-screening locus-combinations with the MS statistic. Each of the four disease loci were involved in multiple interactions that caused risk of multiple latent phenotypes. Additionally, there was heterogeneity across populations in how these latent phenotypes caused the KPD trait. Therefore, the strength of the interaction effects relative to the marginal effects was diluted by the presence of multiple interactions per locus. In this situation the identification of a disease locus is more likely to happen through its marginal effect. The testing framework employed here involved multi-df tests of main effects and interactions, which could lead to positive tests in the presence of main effects but no interaction. However, the number of tests per stage increased with the order of interaction and the alpha level was equally allocated to each of the three stages. This resulted in the test-specific significance threshold increasing with stage, thus a significant multilocus effect test was unlikely to be explained by main effects alone. Also, following Schaid et al. [8] our models assumed log-additive risk and multiplicativity between loci, while the true susceptibility patterns were either dominant or recessive, and multiplicativity did not necessarily hold. Departures from the true risk model may have contributed to our lack of power to detect multilocus effects. For example, various combinations of the three latent phenotypes, P1–P3, determined the KPD trait, and first order interactions, involving dominant and recessive susceptibility patterns, between the four disease regions, D1–D4, influenced risk of P1–P3. Also, the relationship between KPD and P1–P3 varied across the three populations. Therefore, the relationship between D1–D4 and KPD was complicated, and there may not have been adequate information in the data to detect those interactions at the provided sample size. However, it needs to be emphasized that the principle objective of this approach is not to identify interaction effects *per se *but rather to identify loci or combinations of loci that influence disease risk. The method was thus successful in identifying the disease regions through the marginal effects of the SNPs.

## Conclusion

While consistent multilocus effects were not identified by this particular analysis, an approach was documented that simultaneously accounts for possible interactions, maintains adequate power for detecting main effects, and rigorously controls the FDR for multiple tests. A novel method was implemented for jointly testing linkage and association using affected sib-pair-parent data that is computationally fast and easy to implement using standard statistical software. With further research this method could be generalized to nuclear families with more than two affected offspring. Four of the six disease loci were identified in at least a subset of the 10 replicate data sets when all disease-region SNPs were included in the analysis. These results bolster the idea that it is feasible to explicitly account for interactions in a candidate gene study while maintaining adequate power for finding marginal effects.

## Abbreviations

CPG: Conditional-on-parental-genotype

FDR: False discovery rate

GAW14: Genetic Analysis Workshop 14

IBD: Identical by descent

KPD: Kofendrerd personality disorder

LD: Linkage disequilibrium

LRT: Likelihood ratio test

MS: Mating type screening statistic

SNP: Single-nucleotide polymorphism

TDT: Transmission disequilibrium test

## Authors' contributions

The main analytic approach was conceived by JM, who also conducted the data analysis. WJG collaborated in the design of the MS statistic. KDS suggested the use of false discovery rates. KDS, DVC, and WJG participated in regular meetings to discuss the results and suggest further analysis and assisted in the preparation of the final manuscript.

## Figures and Tables

**Table 1:** Number of 10 replicate datasets with significant effects.

SNP			Locus	significant Replicates (Frequency)
Single Locus Effects				
B01T0554			D1	2
B01T0559			D1	2
C01R0052			D1	2
B03T3056			D2	10
B03T3057			D2	10
B03T3058			D2	10
C03R0281			D2	7
B03T3062			D2	1
B03T3063			D2	1
B03T3066			D2	4
B03T3067			D2	6
B05T4135			D3	2
B05T4136			D3	8
C05R0380			D3	7
B05T4138			D3	1
B05T4139			D3	3
B05T4140			D3	2
B05T4141			D3	1
B05T4142			D3	5
B09T8331			D4	4
B09T8332			D4	8
B09T8333			D4	9
B09T8334			D4	4
C09R0765			D4	8
B09T8337			D4	9
B09T8338			D4	5
B09T8339			D4	5
B09T8340			D4	8
B09T8341			D4	9
B09T8342			D4	3
3-Locus Effects				
C01R0052	B03T3056	C05R0380	D1, D2, D3	1
B03T3062	B09T8341	B02T1017	D1, D4, 7	1

## References

[B1] Devlin B, Roeder K, Wasserman L (2003). Analysis of multilocus models of association. Genet Epidemiol.

[B2] Siegmund KD, Gauderman WJ (2001). Association tests in nuclear families. Hum Hered.

[B3] Martin ER, Kaplan NL, Weir BS (1997). Tests for linkage and association in nuclear families. Am J Hum Genet.

[B4] Cordell HJ (2004). Properties of case/pseudocontrol analysis for genetic association studies: effects of recombination, ascertainment, and multiple affected offspring. Genet Epidemiol.

[B5] Millstein J, Seigmund DS, Conti DV, Gauderman WJ (2005). Testing association in the presence of linkage using affected-sib-parent study designs.

[B6] Self SG, Longton G, Kopecky KJ, Liang KY (1991). On estimating HLA/disease association with application to a study of aplastic anemia. Biometrics.

[B7] Schaid DJ, Sommer SS (1993). Genotype relative risks: methods for design and analysis of candidate-gene association studies. Am J Hum Genet.

[B8] Schaid DJ (1996). General score tests for associations of genetic markers with disease using cases and their parents. Genet Epidemiol.

[B9] Weinberg CR (1999). Methods for detection of parent-of-origin effects in genetic studies of case-parents triads. Am J Hum Genet.

[B10] Siegmund KD, Langholz B, Kraft P, Thomas DC (2000). Testing linkage disequilibrium in sibships. Am J Hum Genet.

